# Eighth Annual Meeting of the American Medical Association

**Published:** 1855-07

**Authors:** 


*Eighth Annual Meeting of the American Medical Association.*—We continue our last month's report:

THIRD DAY.

The meeting was called to order punctually at 9 o'clock. The minutes having been read and approved—

The Chair said that there was no regular way of appointing the committees on prize essays, arrangements, &c; it had been usual, however, for the convention to refer the appointment of these committees to the committee of arrangements.

Dr. Watson moved that the subject be referred to that committee on this occasion.

The motion was agreed to.

A letter was read from Dr. Reyburn, resigning his office as chairman of the committee on medical topography, and asking the division of the territory composed of Wisconsin, Missouri, Iowa, and Illinois.

Dr. Watson moved the acceptance of the resignation, and the reference of the other portion of the communication, to the committee on nominations, which was afterward withdrawn.

Dr. Askew renewed the motion, and it was adopted.

Dr. Hays made a personal explanation; after which a letter was read from Dr. E. B. Haskins, of Clarksville, Tenn., chairman of the committee on microscopical investigations on malignant tumours, asking to be excused from making a report, inasmuch as he had not the necessary apparatus for ascertaining the facts pertinent to the subject.

The request of the gentleman was granted.

Dr. Frank H. Hamilton, of Buffalo, continued the reading his report, and at its conclusion he expressed an earnest hope that the surgeons of the Pennsylvania Hospital would hereafter be more accurate in their statistics of fractures; for even from this city of medical science—this Salerno of medical literature, statements have gone forth in relation to an apparatus for dressing broken clavicles, which were inaccurate, and were therefore calculated to do very great injury. He did not wish to speak unkindly, but he besought them to be more careful in future, so that we might not become a grand insurance company for the whole United States of America.

The Hon. Judge Lewis, Chief Justice of the State of Pennsylvania, being present, was unanimously invited to a seat upon the platform.

Dr. J. G. Orton, of New York, offered resolutions requesting medical societies throughout the country to require their members to keep accurate records of their cases, and present annual digests of the same to this association, through the several state societies. Referred to the committee of one from each state.

Dr. Condie, of Pennsylvania, stated that although not quite finished, he was prepared to read his report on "Tuberculous Diseases." It, however, embodied a space of five hundred pages, and an abstract would require sixty pages. He could not expect the association to publish it, but left it entirely with them to do as they pleased, for there would be no difficulty in its being brought out by medical publishers.

On motion, Dr. Condie was authorized to exercise his own discretion in the matter.

On reässembling, Dr. Mussey, of Ohio, read his report upon the "Influence of Alcohol in Health and in Disease." This report was considered highly appropriate to the present period of excitement in regard to the temperance question, and the views of the venerable author seemed to us to coincide fully with those of the advocates for total abstinence. The report was ordered to the publishing committee.

Part of the report by Dr. Blatchford, of New York, on "Hydrophobia," was read by the Secretary, and ordered to be published.

### FOURTH DAY.

The minutes having been read and approved, resolutions of thanks to the committee of arrangements, the Mayor, the guardians of public institutions, and the citizens and physicians of Philadelphia, for courtesies extended to the members, were presented by Dr. Stewart, of New York, and Dr. Hayward, of Massachusetts, and unanimously adopted.

Dr. N. S. Davis, of Illinois, moved the following preamble and resolutions, which were referred to the committee of arrangements, with instructions to report on the same at the commencement of the next annual meeting:

Whereas, The present mode of conducting the annual meetings of the Association affords but little opportunity for the discussion of strictly scientific questions and papers, and whereas this has been regarded as a serious defect in the operation of our organization, impairing its scientific character: therefore,

Resolved, That the daily sessions of the Association during each annual meeting be divided into two parts, the first to terminate at an hour not later than 12½ o'clock, P. M., of each day, and to be devoted as heretofore to the general business of the Association. The second part, consisting of all the time which it is deemed advisable to remain in session each day after 12½ o'clock, P. M., to take the character of a scientific section, and to be devoted exclusively to the discussion of questions relating to the science and art of medicine.

Resolved, That the Association in its capacity of a scientific section, having no power to act on any subject of a scientific character, may continue in session, whenever thought desirable, a longer period than in its more general capacity.

A paper on the subject of religion in connection with medicine, which had been referred to a special committee, was reported upon, and ordered not to be published with the transactions.

Dr. A. J. Semmes, of Washington, D. C., offered the following, which was agreed to:

Resolved, that a committee of three delegates be appointed to report to this association at its next annual meeting, what measures should be adopted to remedy the evils existing in the present method of holding coroner's inquests, by incompetent persons, by which the lives and liberties of the innocent may be jeoparded, and the ends of justice generally frustrated.

Dr. A. J. Atlee, offered the following:

Resolved, That to secure efficient teaching in medical schools, where a prime object is to enforce practical precepts, a large degree of union and harmony must exist among the teachers, and confidence be reposed in them, on the part of their pupils.

Resolved, That any such unnatural union as the mingling of any exclusive system, such as homœopathy, with scientific medicine, in a school, setting aside all questions of its untruthfulness, cannot fail, by the destruction of union and confidence, and the production of confusion and disorder, unsettling the minds of the learners, to so far impair the usefulness of teaching, as to render any school adopting such a policy, unworthy the support of the profession.

This resolution was, we believe, introduced at the request of the representatives of the Medical Department of the University of Michigan. We state this, as the mere wording of the resolution might imply that a censure was intended.

The real object of the resolution was undoubtedly to influence public sentiment in Michigan against the threatened establishment of a Homœopathic chair in that school — a very laudable motive, and one which we hope may result favorably. The policy of such a resolution may, nevertheless, be doubted. It implies a weakness and a need of outside assistance, such as the friends of the institution are unwilling to acknowledge, and unless we are mistaken, will go to confirm the prevalent opinion, that the interests of legitimate medicine in this school, however warmly defended by the present Faculty, are destined to serious injury from the outside pressure of quackery.

The resolutions were unanimously agreed to.

The action of the State Medical Society, of Ohio, in refusing to acquiesce in some of the requirements of the Code of Ethics of the association, was alluded to, and gave rise to an animated discussion; the course of the society was generally condemned, and by none more than by members from Ohio.

Dr. Clendennin offered the following:

Resolved, That no state or local society shall be hereafter entitled to representation in this association, that has not adopted its Code of Ethics.

Resolved, That no state or local society, that has intentionally violated or discarded any article or clause of the Code of Ethics, shall longer be entitled to representation in this body.

A motion to lay the above on the table was lost.

Dr. Miltenberger, of Baltimore, offered a resolution, which was amended by Dr. J. L. Atlee, to read as follows:

Whereas, It has been brought before the notice of the American Medical Association, that the State Medical Society, of Ohio, has violated at their last meeting, sec. 4th, art. 1st, chapter 2d, of its Code of Ethics; therefore be it

Resolved, That the secretary of the association be directed to inform the officers of that society, that unless such action be rescinded, they cannot be hereafter represented in this association.

These, together with the resolutions of Dr. Clendennin, were adopted.

Dr. Corson, of New York, presented and read a volunteer communication on the influence of lead on the heart. The paper was referred to Drs. Davis, Smith, and Stewart, of New York.

Dr. Stille, of Philadelphia, read a series of resolutions proposing to alter the mode of instruction in medical colleges, which were referred to a special committee, to report upon next year.

Numerous amendments to the constitution were proposed, and laid over under the rule, for future consideration. Business having been completed, the association adjourned at 1½ o'clock *sine die*.

During its session, the convention appointed the following committees:

*On Prize Essays.*—Drs. A. B. Palmer, Samuel Denton, A. R. Terry, Adam Sager, S. H. Douglass, C. La Ford, E. Andrews.

*Committee of Arrangements.*—Zina Pitcher, of Detroit, chairman; Moses Gunn, G. B. Russell, A. S. Leland, Morse Stewart, P. Klein, J. A. Brown, all of Michigan.

*Committee on Organization of State and County Societies.*—A. B. Palmer, Detroit; N. B. Ives, Conn.; E. B. Haskins, Tenn.; C. Woodward, Ohio; J. Crosby, N. H.

*Committee on Medical Education.*—W. H. Anderson, Ala.; J. B. Flint, Ky.; P. H. Cabell, Ala.; G. Hayward, Mass.; E. B. Smith, Mo.

*Committee on Medical Literature.*—P. C. Gaillard, S. C.; N. P. Monroe, Maine; J. Couper, Del.; R. Hiles, Ohio; A. Coffin, S. C.

*Committee on Registration of Marriages, Births and Deaths.*—Consisting of one member from each state.

*Committee on Medical Topography, and Epidemics.*—One member from each state.

*Special Committees, to report upon named subjects.*—Lewis H. Steiner, of Washington, D. C., on strychnia—its chemical and texicological properties.

Ashbury Evans, of Covington, Ky., on tracheotomy in epilepsy.

J. Taylor Bradford, of Augusta, Ky., on the treatment of cholera.

Charles Z. Chandler, of Rocheport, Mo., on malignant periodic fevers.

B. A. Johnson, of Chicago, Illinois, on the excretions as an index to the organic changes in the system.

Henry J. Bigelow, of Boston, Mass., on microscopical investigation of malignant tumors.

E. H. Davis, of New York, on the statistics of calculous diseases, and the operations thereof.

J. S. Carpenter, of Pa., on the treatment and curability of reducible hernia.

N. J. Fulder, of Maine, on the best treatment of cholera infantum.

William B. Page, of Philadelphia, on injuries of the joints.

William Jewell, of Philadelphia, on the statistics of mortality in the United States.

J. Knight, of New Haven, Conn., on endemic fevers.

P. H. Cabell, of Ala., on the native substitutes for cincieona, indigenious to the southern states.

James M. Newman, of Buffalo, New York, on the sanitary police of cities.

L. M. Noble, of Le Roy, Ill., on puerperal fever and its communicability.

J. M. Freer, of Chicago, Ill., on the progress of general and descriptive anatomy.

J. M. Corson, of New York, on the causes of the impulse of the heart, and the agencies which influence it in health and disease.

Meredith Reed, of New York, on the causes of infant mortality in large cities, the sources of its increase, and the means for its diminution.

Mark Stephenson, of Vermont, on the treatment best adapted to each variety of cataract, with the method of operation, place of election, time, age, &c.

J. B. Coleman, of New Jersey, on the effect of mercury on the living tissues.

F. G. Richardson, of Louisville, Ky., on the diversity of the venereal poison.

J. B. Flint, of Louisville, Ky., on the best mode of rendering the medical patronage of the national government, tributary to the honor, and improvement, of the profession.

D. M. M. Latta, of Goshen, Indiana, on whether there are any means by which the growth of the fœtus in utero, may be controlled without injury to the mother or child.

Thos. Miller, of Washington, on toxicology.

E. K. Peaslee, of Hanover, N. H., on inflammation, its pathology, and its relation to the reparative process.

D. D. Thompson, of Louisville, Ky., on the remedial effects of chloroform.

Wm. Clendennin, of Cincinnati, Ohio, on epidemic erysipelas.

C. G. Comegys, of Cincinnati, Ohio, on the state of the urine in tubercular disease.

The meeting thus closed, was in many respects a very pleasant one. Good feeling prevailed in the debates, and there was less talking for Buncombe, than is usual. One or two members were, as usual, disagreeably conspicuous, from their propensity to enlighten the association on every subject coming before it. This was a minor evil, however, and was richly counterbalanced by the interesting character of the proceedings in general. The whole number of delegates was over five hundred.

No great dinner was given—a piece of good sense, and good taste, on which we compliment the Philadelphia profession. The private hospitalities of the profession were in excellent taste, and afforded very pleasant gatherings for the evenings. Aside from these, the various excursions to objects of interest, occupied all the spare time of the members, and must have proved no light tax upon the time and efforts of the Committee of Arrangements—a very important committee at such a time, and who in this instance were fully equal to the emergency.

